# Thank you to reviewers 2024

**DOI:** 10.1093/ehjimp/qyaf004

**Published:** 2025-04-24

We wish to thank the following reviewers for their contributions during 2024

**Top reviewers**

Francesco Bianco

Andreas Giannopoulos

Fausto Pizzino

Alberto Aimo

Nicolò De Biase

Sara Moscatelli

Valeria Pergola

Andrea Barison

Abdelrahman Elhakim

Iacopo Fabiani

With thanks to all our 2024 reviewers

**Table:** 

First Name	Last Name
Francesco	Bianco
Fausto	Pizzino
Andreas	Giannopoulos
Alberto	Aimo
Nicolò	De Biase
Sara	Moscatelli
Valeria	Pergola
Iacopo	Fabiani
Julia	Grapsa
Abdelrahman	Elhakim
Andrea	Barison
Lavinia	Del Punta
Giancarlo	Trimarchi
Pieter	van der Bijl
Georgios	Benetos
Federico	Fortuni
Edoardo	Conte
Giuseppe	Ciliberti
Stefano	Albani
Pietro	Scicchitano
Soumaya	Sridi-Cheniti
Kyriakos	Yiangou
Amalia	Peix
Rebecca	Perry
Elena	Galli
Erika	Hutt
Erik Andreas	Berg
Konstantinos	Papadopoulos
Riccardo	Liga
Nunzia	Borrelli
Marta	Cvijic
Konstantinos	Katogiannis
Krasimira	Hristova
Lucia	La Mura
Jolanda	Sabatino
Shahab	Masoumi
Michele	Coceani
Maribel	Gonzalez del Hoyo
Andrea	Barbieri
Paolo	Morfino
Fabrizio	Ricci
Ivan	Stankovich
Edoardo	Zancanaro
Claudio Tinoco	Mesquita
David	Prior
Chrysanthos	Grigoratos
Chris	Roy
Tiffany Dong	Dong
Christoph	Nienaber
Ana	Almeida
Angelica	Cersosimo
Vincenzo	Nuzzi
Leyla Elif	Sade
Valentina	Barletta
Stefano	Figliozzi
Rosaria	Barracano
Simona	Celi
Ruud	Van Heeswijk
Stéphane	Manzo-Silberman
Shehab	Anwer
Thomas	MD
Yu Fu	Ferrari Chen
Vlatka	Reskovic
George	E Wesbey MD
William	Watson
Anna	Sannino
Antonella	Meloni
Arash	Forodighasemabadi
Aldo	Schenone
Andrea	Baggiano
Ana	Timoteo
Ambreen	Ali
Lamia	Ait Ali
Anastasia	Kitsiou
Attila	Kovács
Cian	Scannell
Blanche	Cupido
Blazej	Michalski
Faiza	Al Kindi
Valerio	Di Fiore
Dimitrios	Vlastos
Dan-Mihai	Dorobantu
Dominik	Benz
Dominik	Sager
Rahul	Chaudhary
Filippo	Zilio
Erik	Hedström
Erwan	Donal
Humberto	Morais
Matteo	Parollo
Paul	Cremer
Pauline	Gut
Marco	Guglielmo
Magnus	Bäck
Mouaz	Al Mallah
Margarita	Brida
Carolina	Brás
Jing	Liu
Konstantinos	Moulakakis
Polona	Kacar
Olivier	Lairez
Laust Dupont	Rasmussen
Leah	Wright
Leslee J	Shaw
Lino	Gonçalves
Lilit	Baghdasaryan
Jon Magne	Letnes
Jürgen	Duchenne
Konstantinos	Vakalis
AIKATERINI	KOUNTOURI
Simrat	Kaur
Jah Yeon	Choi
Alessia	Gimelli
Giulia Elena	Mandoli
Mazen	Hanna
Ignatios	Ikonomidis
Isabella	Leo
ISRAEL	VALVERDE
Wael	Jaber
Jamal	Khan
Jan	Verwerft
Jerome	Garot
Per	Arvidsson
Ronny	Buechel
Patrick	Doeblin
Pietro	Costantini
Vincenzo	Positano
Prasanna	Venkataraman
Regina	Bokenkamp
Rozemarijn	Vliegenhart
Rasmus	Paulsen
Rhanderson	Cardoso
Rami	Doukky
Ricardo	Ladeiras-Lopes
OUNG	Savly
Panagiota	Mitropoulou
Paolo	Frumento
Hideharu	Oka
Nina	Hasselberg
Mihaly	Karolyi
Manuel	Morales
Monica	Deac
Nausheen	Akhter
Matthias	Eberhard
Maryam	Alsharqi
fatima	ezzahra
Federico	Caobelli
Felipe	Sanchez Tijmes
Filipa	Valente
Emmanuel	Androulakis
Enrica	Vitale
Giulia	De Zan
Wensu	Chen
Chiara	Caselli
David	Sulman
Eirini	Beneki
Chiara	Coletti
Brett	Sperry
Carmelo	De Gori
In-Cheol	Kim
Bernhard	Föllmer
Brian	Hoit
Aju	Pazhenkottil
Alessandra	Scatteia
Andreina	D'Agostino
Amer	Barakat
Andrew	Einstein
Angela	Pucci
Ahmad	Sayed
Tom	Wang
Serkan	Ãœnlüs
Vinesh	Appadurai
Valeria	De Luca
Satoshi	Nakamura
Efstathios	Pagourelias
Svetlin	Tsonev
Athanasios	Rempakos
Stefano	Sforna
Stefano	Nistri
Rosa	Sicari
Nobuaki	Kobayashi
Sara	Bombace

## Notes

**Funding:** There is no funding for this article.

